# Prevalence and diagnostic value of non-criteria antiphospholipid antibodies for antiphospholipid syndrome in Chinese patients

**DOI:** 10.3389/fimmu.2023.1107510

**Published:** 2023-04-12

**Authors:** Siting Li, Yina Bai, Jingjing Meng, Qian Wang, Xinping Tian, Mengtao Li, Xiaofeng Zeng, Jiuliang Zhao, Chaojun Hu

**Affiliations:** ^1^ Department of Rheumatology and Clinical Immunology, Peking Union Medical College Hospital, Chinese Academy of Medical Sciences, Peking Union Medical College, National Clinical Research Center for Dermatologic and Immunologic Diseases (NCRC-DID), Ministry of Science & Technology, State Key Laboratory of Complex Severe and Rare Diseases, Peking Union Medical College Hospital (PUMCH), Key Laboratory of Rheumatology and Clinical Immunology, Ministry of Education, Beijing, China; ^2^ Department of Clinical Laboratory, Fifth Affiliated Hospital of Zhengzhou University, Zhengzhou, China

**Keywords:** antiphospholipid antibody syndrome, non-criteria antiphospholipid antibody, extra-criteria manifestation, thrombosis, pregnancy morbidity

## Abstract

**Background:**

The presence of antiphospholipid antibodies (aPLs) plays a pivotal role in the pathogenesis of antiphospholipid antibody syndrome (APS). This study aimed to examine the diagnostic value of a set of non−criteria aPLs and their relevance with APS-related criteria and extra-criteria manifestations.

**Methods:**

From a prospectively constructed database, consecutive APS patients consisting of 114 primary APS (PAPS group), 54 with APS secondary to SLE (SAPS group), 9 seronegative APS (SNAPS), as well as 209 patients with systemic lupus erythematosus (SLE) and 88 healthy controls were included in this study. Levels of criteria aPLs, baseline information, and APS-related criteria and extra-criteria features were extracted from the database. Serum levels of non-criteria aPLs including aPC IgG/IgM, aPI IgG/IgM, aPE IgG/IgM/IgA, aPG IgG/IgM/IgA, anti-phosphatidic acid (aPA) IgG/IgM, aSM IgG/IgM, and aPS/PT IgG/IgM were analyzed with AESKULISA® ELISA Test Kits.

**Results:**

The addition of aPC IgG/M, aPI IgG/M, aPE IgG/M/A, aSM IgG/M, and aPA IgG/M to aCL or aβ2GPI IgG/M could significantly increase diagnostic sensitivity and accuracy. A significant difference between PAPS or SAPS and HC was presented in all non-criteria aPLs except for aSM IgM and aPG IgA. Eight out of nine SNAPS patients were positive for at least 1 aPL. Pregnancy morbidity was associated with aSM IgM (r = 0.22) and aSM IgG (r = 0.15). Pre-eclampsia or premature birth was associated with aSM IgG (r = 0.16), aPI IgG (r = 0.22), aPC IgG (r = 0.16), and aPG IgG (r = 0.18). Stroke was associated with aPI IgG (r = 0.2). The clinical association was also observed in DVT with aPS/PT IgG (r = 0.17). Valve lesion was positively associated with aSM IgM (Fisher test p = 0.039), APS nephropathy was associated with aPC IgG (OR 3.797), and livedo reticularis was associated with aPE IgM (OR 15.391).

**Conclusion:**

Additional detection of non-criteria aPLs including aPC IgG/M, aPE IgG/M/A, aPI IgG/M, aSM IgG/M, and aPA IgG/M could assist in APS diagnosis. The positivity of certain aPLs was statistically associated with both criteria and extra-criteria APS clinical manifestations.

## Introduction

Antiphospholipid syndrome (APS) is characterized by thrombosis and/or pregnancy morbidity with the persistent presence of high antiphospholipid antibodies (aPLs), which contribute significantly to the disease’s pathogenesis. Golden classification criteria for APS (Sydney criteria) required the presence of at least one clinical criteria as well as one of the laboratory criteria including lupus anticoagulant (LA), medium to high level of anti-cardiolipin (aCL), anti-β2 glycoprotein-I (aβ2GPI) immunoglobulin isotype G (IgG) or M (IgM) positivity at 12 weeks apart (1). In real-world clinical scenarios, patients may have positive laboratory results of unclear clinical significance (2), whereas others (known as seronegative APS, SNAPS) are present with clinical manifestations highly suggestive of APS but persistently negative for standard aPLs (3).

Among new biomarkers investigated for APS, non-criteria aPLs such as anti-β2GPI domain I (aDM1), anti-phosphatidylserine/prothrombin (aPS/PT), anti-phosphatidic acid (aPA), and anti- phosphatidylinositol (aPI) is increasingly recognized (4). Besides diagnosis, evaluation of non-criteria aPLs could also contribute to prognosis and risk assessment for associated clinical manifestations (5). However, due to the heterogeneity of detection methods and population, the diagnostic value of these aPLs remains controversial.

Regarding the Chinese population, previous studies indicated that aPS/PT could identify some SNAPS patients and was associated with thrombotic and obstetric complications (6, 7). Our previous work in a smaller cohort suggested that IgG or IgM antibodies of phosphatidylserine (aPS), aPI, sphingomyelin (aSM), phosphatidylcholine (aPC) and phosphatidylethanolamine (aPE) were helpful for identifying SNAPS and predicting arterial thrombosis (8). Few studies have explored all of the aforementioned extra-criteria autoantibodies, and their relations with more detailed clinical manifestations in the same patient groups. This study utilized commercial ELISA kits to test the levels of 16 aPLs in APS patients and disease or healthy controls. The diagnostic value and clinical relevance of each aPL isotype were further investigated.

## Materials and methods

### Patient groups

This was a single-center study conducted at Peking Union Medical College Hospital (PUMCH). Starting in March 2010, a database of patients with rheumatic diseases including APS and SLE was prospectively constructed at the Chinese Rheumatism Data Center at PUMCH. APS was diagnosed according to the 2006 Sydney revised classification criteria (1), and SLE was diagnosed using the 1997 ACR criteria (9). From 2009 to 2021, a total of 177 consecutive APS outpatient cases from the database were included in this study, of which 114 patients had been diagnosed with primary APS (PAPS group), 54 with APS secondary to SLE (SAPS group), and 9 were clinically diagnosed with seronegative APS (SNAPS). Totally 201 SLE patients (SLE group) and 88 healthy controls (HC group) were also included for analysis. Patients with vasculitis were excluded from the study.

Upon diagnosis, serum samples were collected at the outpatient clinic and immediately analyzed for aPL antibodies at the Key Laboratory of the Department of Rheumatology, PUMCH. Besides aPL serology, baseline information including history clinical manifestations, ANA positivity, and current medication was collected. Thrombosis (arterial or venous), pregnancy morbidity, and extra-criteria manifestations were defined according to the classification criteria (1). For the HC group, only aPL serology information was present. The study was approved by the ethics committee at PUMCH and fulfilled the ethical guidelines of the declaration of Helsinki. All subjects gave written informed consent.

### Antibody and laboratory tests

IgG and IgM isotypes of aCL and anti-β2GPI for each study subject were analyzed with QUANTA Flash® CLIA kits provided by INOVA Diagnostics, Inc. The cutoff value was defined as 24 U/ml as recommended by the manufacturer. Lupus anticoagulant was detected and evaluated according to the ISTH recommendations. Dilute Russell viper venom time (dRVVT) testing and activated partial thromboplastin time were measured, and LAC was considered positive if the ratio of the screen/confirm time ratio was >1.20. Non-criteria aPLs including aPC IgG/IgM, aPI IgG/IgM, aPE IgG/IgM/IgA, aPG IgG/IgM/IgA, anti-phosphatidic acid (aPA) IgG/IgM, aSM IgG/IgM, and aPS/PT IgG/IgM were analyzed with AESKULISA® ELISA Test Kits provided by Aesku. Diagnostics GmbH & Co. KG (Wendelsheim, Germany). Cut-off values for aPC, aPI, aPE, aPG, aPA, and aSM were calculated with the 95.5% percentile of test levels from 88 healthy controls since the distribution was not normal. Cut-off values for PSPT IgG/M were defined as 30 U/mL as recommended by the manufacturer.

### Statistical analysis

Statistical analysis was performed using SPSS 26.0 or R (version 4.0.2). Sensitivities, specificities, and accuracies in APS diagnosis were compared using the McNemar test. The Youden Index, positive and negative predictive values (PPV and NPV), and odds ratio (OR) with 95% confidence intervals (95% CI) were also shown. The χ2 test or Fisher’s exact test was used for the comparison of categorical variables, and the Wilcoxon test was used for continuous variables after normality was explored with the Shapiro-Wilk test. Pearson correlation and complete-link cluster methods were used to explore the relationship between aPLs and diagnostic clinical manifestations. Associations between non-criteria aPLs isotype positivity and extra-criteria clinical manifestations in patients with APS were calculated with odds ratio (OR) and displayed in 95% CI. Two-tailed values of p less than 0.05 were considered statistically significant.

## Results

### Patient characteristics

Among all subjects included, there were 72(63.2%) females for PAPS, 47 (87.0%) for SAPS, 9 (100%) for SNAPS, 192 (91.9%) for SLE, and 70 (79.5%) for HCs (**Table 1**). The mean age was 37.2 ± 10.8 years for PAPS, 36.0 ± 9.9 years for SAPS, 32.2 ± 5.4 years for SNAPS, 34.2 ± 9.4 years for SLE, and 42.5 ± 12.1 years for HC. Clinical manifestations were selectively shown for APS and SLE patients. Pregnancy morbidity was present in 51.4% (37/114) of PAPS, 44.7% (21/54) of SAPS, and 66.7% (6/9) of SNAPS patients, while only 2.9% (6/209) of SLE patients met the definition. History of arterial events was present in 35.1% (30/114) of PAPS, 29.6% (16/54) of SAPS, and 11.1% (1/9) of SNAPS patients, whereas venous events were present in 52.6% (60/114) of PAPS, 40.7% (22/54) of SAPS, and 33.3% (3/9) of SNAPS patients. No thrombotic event was observed for patients with only SLE. Most of the events that occurred were stroke (21.1% in PAPS, 16.7% in SAPS, and 11.1% in SNAPS) and deep vein thrombosis (38.6% in PAPS and 33.3% in SAPS). Regarding medication, 70 (61.4%) PAPS, 36 (66.7%) SPAS, 9 (100%) SNAPS, and 55 (26.3%) SLE patients had taken antiplatelet medicines. There were 91 (79.8%) PAPS, 40 (74.1%) SAPS, 2 (22.2%) SNAPS, and 8 (3.8%) SLE patients taking anticoagulants, and 106 (93%) PAPS, 52 (96.3%) SAPS, 7 (77.8%) SNAPS, and 165 (78.9%) SLE patients on hydroxychloroquine. In addition, 41 (36.0%) PAPS, 48 (88.9%) SPAS, 5 (55.6%) SNAPS, and 160 (76.6%) SLE patients received glucocorticoid therapy upon inclusion.

**Table 1:** Baseline information for APS, SLE, and healthy controls (n = 474).

	APS (177)			SLE (209)	Healthy controls (88)
	PAPS (114)	SAPS (54)	SNAPS (9)		
Gender (female/male)	72/42	47/7	9/0	192/17	70/18
Mean age (mean years ± SD)	37.2 ± 10.8	36.0 ± 9.9	32.2 ± 5.4	34.2 ± 9.4	42.5 ± 12.1
BMI (mean kg/m^2^ ± SD)	24.3 ± 3.7	24.6 ± 4.7	22.0 ± 4.4	22.5 ± 3.5	NA
ANA, n (%)	32 (28.1)	53 (98.1)	5 (55.6)	202 (96.7)	0
Coombs, n (%)	14 (12.3%)	30 (55.6%)	2 (22.2%)	77 (36.8%)	NA
APS-related clinical manifestations					
Pregnancy morbidity, n (female%)	37 (51.4%)	21(44.7%)	6 (66.7%)	6 (2.9%)	NA
Early embryonic loss (<10W ≥1), n (%)	14 (19.4%)	11(23.4%)	1 (11.1%)	6 (2.9%)	NA
Consecutive embryonic loss (<10W ≥3), n (%)	3 (4.2%)	0	0	0	NA
Late fetal loss (10-28 W), n (%)	23 (31.9%)	14 (29.8%)	3 (33.3%)	2 (1.0%)	NA
Premature birth, n (%)	13 (18.1%)	9 (19.1%)	3 (33.3%)	4 (1.9%)	NA
History of arterial events, n (%)	40 (35.1%)	16 (29.6%)	1 (11.1%)	0	NA
Stroke, n (%)	24 (21.1%)	9 (16.7%)	1 (11.1%)	0	NA
Coronary heart disease, n (%)	6 (5.3%)	3 (5.6%)	0	0	NA
Eye involvement, n (%)	5 (4.4%)	1 (1.9%)	0	0	NA
Lower limb artery occlusion, n (%)	8 (7%)	0	0	0	NA
Adrenal artery thrombosis, n (%)	0	1 (1.9%)	0	0	NA
TIA, n (%)	3 (2.6%)	0	0	0	NA
History of venous events, n (%)	60 (52.6%)	22 (40.7%)	3 (33.3%)	0	NA
Deep vein thrombosis, n (%)	44 (38.6%)	18 (33.3%)	0	0	NA
Pulmonary embolism/CTEPH, n (%)	27 (23.7%)	15 (27.8%)	0	0	NA
Portal vein thrombosis, n (%)	5 (4.4%)	0	1(11.1%)	0	NA
Cerebral venous and sinus thrombosis, n (%)	8 (7%)	1 (1.9%)	1(11.1%)	0	NA
Central retinal venous occlusion, n (%)	1 (0.9%)		1(11.1%)	0	NA
Superficial venous thrombosis, n (%)	1 (0.9%)	1 (1.9%)	0	0	NA
Microangiopathy, n (%)	13 (11.4%)	19 (35.2%)	0	0	NA
Non-stroke CNS manifestations, n (%)	4 (3.5%)	10 (18.5%)	0	NA	NA
White matter lesions, n (%)	1 (0.9%)	1 (1.9%)	0	NA	NA
Heat valve disease, n (%)	0	12 (22.2%)	0	NA	NA
Antiphospholipid syndrome nephropathy, n (%)	8 (7%)	4 (7.4%)	0	NA	NA
Livedo reticularis, n (%)	2 (1.8%)	3 (5.6%)	0	NA	NA
Bone infarction, n (%)	1 (0.9%)	1 (1.9%)	0	NA	NA
Hematological disorder, n (%)					
Thrombocytopenia, n (%)	35 (30.7%)	32 (59.3%)	1 (11.1%)	63 (30.1%)	NA
Autoimmune hemolytic anemia, n (%)	5 (4.4%)	13 (24.1%)	2 (22.2%)	7 (3.3%)	NA
Medication					
Antiplatelet	70 (61.4%)	36 (66.7%)	9 (100%)	55 (26.3%)	NA
anticoagulant	91 (79.8%)	40 (74.1%)	2 (22.2%)	8 (3.8%)	NA
GC	41 (36.0%)	48 (88.9%)	5 (55.6%)	160 (76.6%)	NA
HCQ	106 (93%)	52(96.3%)	7 (77.8%)	165 (78.9%)	NA
IVIG	1 (0.9%)	7 (13%)	0	3 (1.4%)	NA
AZA	8 (7%)	10 (18.5%)	1 (11.1%)	19 (9.1%)	NA
CTX	9 (7.9%)	10 (18.5%)	1 (11.1%)	8 (3.8%)	NA
CsA	2 (1.8%)	0	0	5 (2.4%)	NA
MTX	0	0	0	13 (6.2%)	NA
MMF	5 (4.4%)	15 (27.8%)	1 (11.1%)	59 (28.2%)	NA
GTW	0	1 (1.9%)	0	1 (0.5%)	NA
TAC	8 (7%)	14 (25.9%)	1 (11.1%)	45 (21.5%)	NA
SRL	14 (12.3%)	3 (5.6%)	0	10 (4.8%)	NA
TGP	1 (0.9%)	0	0	1 (0.5%)	NA

BMI, body mass index; ANA, antinuclear antibodies; CTEPH, chronic thromboembolic pulmonary hypertension; CNS, central nervous system; GC, glucocorticoid; HCQ, hydroxychloroquine; IVIG, intravenous immunoglobulin; AZA, 5-Azacytidine; CTX, cyclophosphamide; CsA, cyclosporin A; MTX, methotrexate; MMF, mycophenolate mofetil; GTW, tripterygium wilfordii multiglycoside; TAC, tacrolimus; SRL, sirolimus; TGP, paeony. NA, not available.

### Predictive value of aPLs in APS diagnosis

The diagnostic power of LA and aPLs positivity was evaluated for sensitivity, specificity, accuracy, Youden Index, positive predictive value (PPV), negative predictive value (NPV), positive and negative likelihood ratio (LR), and odds ratio (OR) in APS diagnosis from HC group in **Table 2** (n = 265). Cutoff values for each aPL were also listed. Sensitivities for LA, aCL, and aβ2GpI were 80.2%, 58.2%, and 70.6%, while the specificity of which were all 100%. For each non-criteria aPL, the sensitivity and accuracy of the combination of aPL IgG, IgM, or IgA were compared to that of aCL or aβ2GpI IgG or IgM. The result indicated that the addition of aPC IgG/M, aPI IgG/M, aPE IgG/M/A, aSM IgG/M, and aPA IgG/M to aCL or aβ2GPI IgG/M could significantly increase diagnostic sensitivity and accuracy. For aPG IgG/M/A and aPS/PT IgG/M, despite significant improvement of sensitivity, accuracy decreased because of loss of specificity.

**Table 2:** Predictive value of aPLs for APS patients.

	Cutoff values^a^	Sensitivity (%)	Specificity (%)	Accuracy (%)	Youden Index	PPV (%)	NPV (%)	LR+	LR-	OR
LA	NA	80.2	100	86.8	0.802	100	71.5	∞	0.198	∞
aCL IgG/M	24	58.2	100	72.1	0.582	100	54.3	∞	0.418	∞
aβ2GpI IgG/M	24	70.6	100	80.4	0.706	100	62.9	∞	0.294	∞
aCL or aβ2GpI IgG/M	24	75.1	100	83.4	0.751	100	66.7	∞	0.249	∞
aPC IgG	2.968	38.4	95.5	57.4	0.339	94.4	43.5	8.533	0.645	8.452(3.187-22.418)
aPC IgM	2.980	31.1	92.0	51.4	0.231	88.7	39.9	3.888	0.749	3.906 (1.857-8.219)
aPC IgG/M	NA	54.8	87.5	65.7	0.423	89.8	49.0	4.384	0.517	4.384 (2.482-7743)
aCL or aβ2GpI or aPC IgG/M	NA	81.9#	87.5	83.8*	0.694	92.9	70.6	6.552	0.207	6.554(3.754-11.440)
aPI IgG	4.755	58.8	95.5	70.9	0.543	96.3	53.5	13.067	0.431	12.927 (4.923-33.942)
aPI IgM	4.440	62.1	95.5	73.2	0.576	96.5	55.6	13.800	0.397	13.672(5.212-35.863)
aPI IgG/M	NA	54.3	92.0	84.9	0.463	95.4	71.1	6.788	0.497	10.228(5.007-20.891)
aCL or aβ2GpI or aPI IgG/M	NA	88.1#	92.0	89.5*	0.801	95.7	79.4	11.013	0.129	11.080(5.432-22.599)
aPE IgG	5.487	40.1	95.5	58.5	0.356	94.7	44.2	8.911	0.627	8.825(3.331-23.378)
aPE IgM	53.934	23.7	95.5	47.5	0.192	91.3	38.4	5.267	0.799	5.220(1.934-14.094)
aPE IgA	19.576	11.9	95.5	39.6	0.074	84.0	35.0	2.644	0.923	2.610(0.924-7.372)
aPE IgG/M/A	NA	53.7	87.5	64.9	0.412	89.6	48.4	4.296	0.529	4.292(2.430-7.589)
aCL or aβ2GpI IgG/M or aPE IgG/M/A	NA	84.2#	87.5	85.3*	0.717	93.1	73.3	6.736	0.181	6.734(3.860-11.748)
aPG IgG	3.365	31.6	95.5	52.8	0.271	93.3	41.0	7.022	0.716	6.960(2.608-18.576)
aPG IgM	2.668	33.9	93.2	53.5	0.271	90.9	41.2	4.985	0.709	4.972(2.235-11.057)
aPG IgA	13.739	7.3	95.5	36.6	0.028	76.5	33.9	1.622	0.971	1.616(0.543-4.811)
aPG IgG/M/A	NA	53.1	85.2	63.8	0.383	87.9	47.5	3.588	0.550	3.595(2.136-6.050)
aCL or aβ2GpI IgG/M or aPG IgG/M/A	NA	80.2#	85.2	81.8	0.654	91.6	68.2	5.419	0.232	5.431(3.270-9.018)
aSM IgG	23.628	27.1	95.5	49.8	0.226	92.3	39.4	6.022	0.763	5.966(2.223-16.015)
aSM IgM	53.974	24.9	95.5	48.3	0.204	91.7	38.7	5.533	0.786	5.469(2.020-14.735)
aSM IgG/M	NA	36.7	93.2	55.4	0.299	91.5	42.3	5.397	0.679	5.386(2.429-11.942)
aCL or aβ2GpI or aSM IgG/M	NA	81.9#	93.2	85.6*	0.751	96.0	71.9	12.044	0.194	12.015(5.533-26.093)
aPA IgG	1.942	71.2	95.5	79.2	0.667	96.9	62.2	15.822	0.302	15.661(5.984-40.985)
aPA IgM	4.187	39.5	95.5	58.1	0.35	94.6	44.0	8.778	0.634	8.701(3.283-23.058)
aPA IgG/M	NA	79.7	92.0	83.8	0.717	95.3	69.2	9.963	0.221	10.015(4.901-20.464)
aCL or aβ2GpI or aPA IgG/M	NA	89.3#	92.0	90.2*	0.813	95.8	81.0	11.163	0.116	11.222(5.503-22.884)
aPS/PT IgG	20	32.8	72.7	46.1	0.055	70.7	35.0	1.201	0.924	1.202(0.804-1.795)
aPS/PT IgM	20	53.7	68.2	58.4	0.219	77.2	42.3	1.689	0.679	1.687(1.207-2.358)
aPS/PT IgG/M	NA	59.3	48.9	55.8	0.082	70.0	37.4	1.160	0.832	1.160(0.914-1.472)
aCL or aβ2GpI or aPS/PT IgG/M	NA	84.2#	48.9	72.4	0.331	76.8	60.6	1.648	0.323	1.646(1.329-2.039)

a Cutoff value was calculated with 95.5% percentile of healthy controls or recommended by manufacturers.

# Significant higher sensitivity compared to the result of aCL or aB2GpI IgG/M (sensitivity 75.1%).

* Significant higher accuracy compared to the result of aCL or aB2GpI IgG/M (accuracy 83.4%s).

PPV, positive predictive value; NPV, negative predictive value; LR, likelihood ratio; OR, odds ratio. NA, not applicable.

### Distribution of antiphospholipid antibodies in different groups

**Figure 1:**
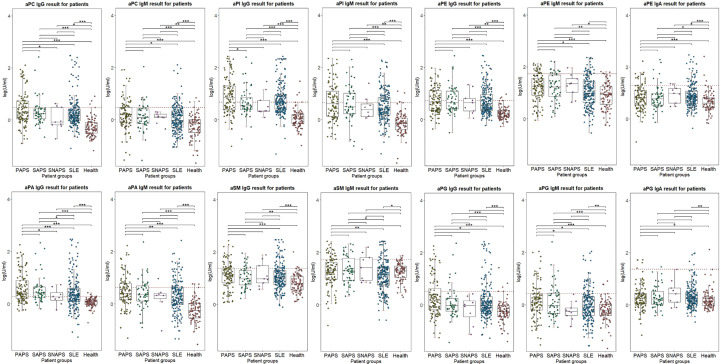
Distribution of aPLs in different patient groups. Log(test levels) was used for illustration. Cutoff value was indicated with red dotted lines. ***p < 0.001; **p < 0.01; *p < 0.05.

The distribution of all criteria or non-criteria aPLs among different patient groups was illustrated in **Figure 1**. Levels of aPLs were calculated with (log (test value) U/ml). A significant difference between PAPS or SAPS and HC was presented in all non-criteria aPLs except for aSM IgM and aPG IgA. Regarding SNAPS patients, significantly different levels of aPC IgG, aPC IgM, aPI IgG, aPI IgM, aPE IgG, aPE IgM, and aPE IgA were observed compared to HC. For differential diagnosis, significantly higher levels of aPA IgG and aSM IgM were present for SAPS patients compared to patients with only SLE.

### Positivity of non-criteria aPLs in the different clinical groups

**Figure 2:**
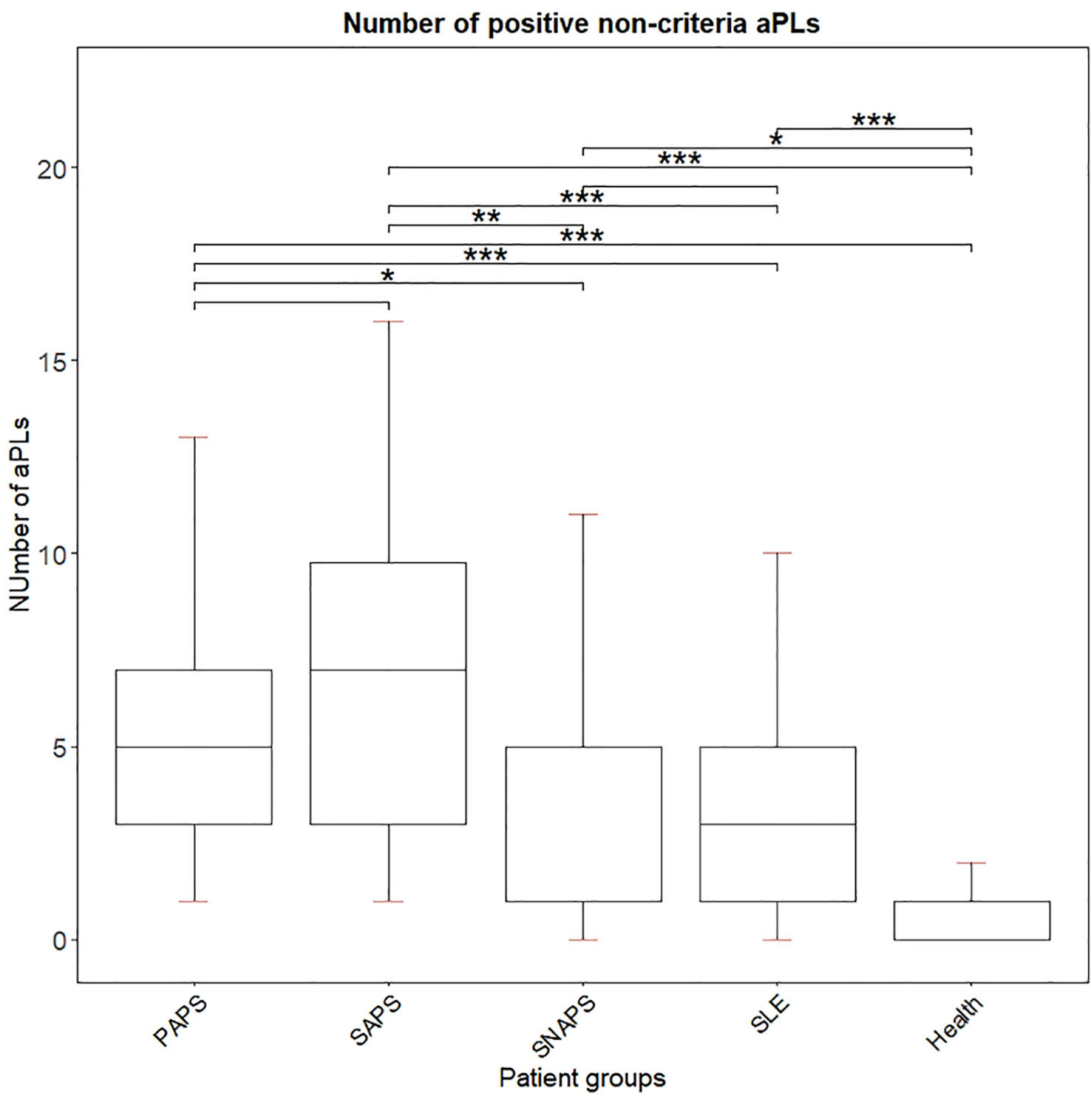
Number of positive non-criteria aPLs for each disease group. ***p < 0.001; **p < 0.01; *p < 0.05.

**Figure 2** demonstrated the number of positive non-criteria aPLs in the different clinical groups. Patients from the PAPS and SAPS groups had a significantly higher number of positive aPLs compared to other groups, and all four disease groups had significantly more aPLs positivity compared to healthy subjects.

For 9 SNAPS patients, the number of positive non-criteria aPLs was listed in **Supplemental Figure 1**. Eight out of 9 SNAPS patients were positive for at least 1 aPLs, of which 1 patient had 11 positive non-criteria aPLs, 2 patients had 5 positive aPLs, and 5 patients had 1 positive aPLs. The aPLs appeared most in SNAPS patients were aPS/PT IgM (in 6 patients), aPI IgG (in 5 patients), and aPA IgG (in 4 patients).

### Correlation of aPLs with criteria and extra-criteria clinical manifestations

Correlations between extra-criteria manifestations and aPLs in APS patients were calculated with odds ratio in **Table 3**. Stroke was significantly associated with aPC IgG (OR 2.272, 95% CI 1.041-4.957), aPG IgG (OR 2.897, 95% CI 1.311-6.400), aPA IgG (OR 3.026, 95% CI 1.101-5.848), and aPS/PT IgM (OR 2.537, 95% CI 1.101-5.848). The late embryonic loss was significantly associated with aPI IgM (OR 2.894, 95% CI 1.225-6.835).

**Table 3:** Clinical correlations between non-criteria aPLs and criteria clinical manifestations (n = 177).

	Arterial events				Venous events				Pregnancy morbidity		
	stroke	coronary heart disease	eye involvement	lower limb artery occlusion	deep vein thrombosis	pulmonary embolism	portal vein thrombosis	cerebral venous and sinus thrombosis	early embryonic loss	late embryonic loss	premature birth/pre-eclampsia
aCL IgG/M	**2.537(1.101-5.848)**	0.469(0.110-1.993)	0.402(0.071-2.283)	**1.143(1.068-21.388)**	0.938(0.500-1.759)	1.552(0.760-3.169)	0.386(0.064-2.324)	1.463(0.388-5.522)	1.828(0.727-4.599)	1.163(0.539-2.509)	**2.873(1.057-7.808)**
aβ2GpI IgG/M	1.866(0.82804.206)	1.301(0.293-5.776)	0.878(0.150-5.141)	0.844(0.150-4.739)	1.603(0.821-3.131)	1.510(0.727-3.135)	0.684(0.109-4.306)	0.528(0.105-2.651)	1.590(0.654-3.867)	0.490(0.210-1.142)	1.392(0.562-3.449)
LA	1.494(0.464-4.810)	1.442(0.476-8.209)	Fisher p = 0.600	Fisher p = 0.359	**11.278(0.2560-49.672)**	**Fisher p < 0.001**	0.450(0.034-5.918)	0.746(0.134-4.141)	3.251(0.903-11.695)	0.364(0.157-0.843)	0.720(0.276-1.879)
aPC IgG	**2.272(1.041-4.957)**	0.460(0.91-2.317)	0.839(0.148-4.753)	1.122(0.247-5.094)	1.159(0.611-2.198)	1.300(0.641-2.638)	0.3317(0.035-2.896)	1.106(0.298-4.105)	0.688(0.272-1.739)	0.962(0.438-2.113)	1.533(0.619-3.792)
aPC IgM	1.982(0.889-4.418)	0.672(0.133-3.409)	0.436(0.049-3.857)	0.356(0.042-3.033)	1.021(0.519-2.009)	0.735(0.337-1.603)	2.827(0.510-15.671)	1.007(0.247-4.098)	1.896(0.786-4.576)	0.766(0.340-1.727)	1.337(0.542-3.299)
aPI IgG	2.037(0.888-4.671)	0.564(0.144-2.210)	0.712(0.139-3.653)	0.755(0.177-3.223)	1.496(0.785-2.851)	1.568(0.757-3.248)	0.365(0.062-2.1440	0.704(0.195-2.541)	0.825(0.343-1.988)	0.664(0.307-1.434)	1.904(0.728-4.979)
aPI IgM	0.923(0.419-2.033)	0.399(0.099-1.616)	0.307(0.054-1.736)	0.266(0.055-1.278)	0.833(0.439-1.583)	1.006(0.491-2.059)	1.563(0.258-9.467)	0.865(0.231-3.231)	0.631(0.263-1.514)	**2.894(1.225-6.835)**	1.741(0.666-4.553)
aPE IgG	1.445(0.658-3.171)	0.458(0.090-2.321)	1.610(0.305-8.508)	2.099(0.474-9.296)	1.103(0.580-2.098)	1.027(0.502-2.098)	0.397(0.043-3.699)	1.094(0.291-4.114)	1.555(0.653-3.703)	0.580(0.268-1.258)	1.409(0.585-3.397)
aPE IgM	1.953(0.802-4.755)	2.385(0.514-11.056)	0.602(0.066-5.470)	Fisher p = 0.201	0.645(0.294-1.419)	0.674(0.280-1.622)	0.781(0.082-7.463)	0.932(0.182-4.778)	2.286(0.919-5.684)	1.091(0.474-2.514)	1.577(0.618-4.025)
aPE IgA	0.424(0.089-.2.014)	0.887(0.096-8.217)	Fisher p = 1.000	1.442(0.145-14.353)	1.300(0.487-3.468)	1.112(0.366-3.374)	Fisher p = 1.000	0.842(0.094-7.542)	0.663(0.167-2.626)	0.928(0.288-2.985)	1.320(0.368-4.735)
aPG IgG	**2.897(1.311-6.400)**	0.622(0.123-3.136)	0.447(0.051-3.948)	1.498(0.331-6.790)	1.196(0.614-2.329)	1.129(0.538-2.368)	0.484(0.052-4.458)	0.531(0.108-2.599)	1091(0.432-2.754)	0.692(0.300-1.600)	2.309(0.928-5.746)
aPG IgM	0.619(0.230-0.544)	0.559(0.494-2.869)	0.406(0.045-3.643)	0.304(0.035-2.642)	1.082(0.555-2.111)	0.857(0.403-1.823)	3.587(0.604-21.319)	0.880(0.214-3.621)	1.451(0.607-3.472)	0.966(0.443-2.106)	1.902(0.784-4.614)
aPG IgA	1.157(0.277-4.837)	Fisher p = 1.000	Fisher p = 1.000	1.597(0.152-16.777)	0.78(0.221-2.784)	0.266(0.033-2.165)	Fisher p = 1.000	1.303(0.139-12.237)	Fisher p = 0.221	0.526(0.104-2.661)	1.880(0.433-8.161)
aSM IgG	1.592(0.686-3.694)	0.371(0.044-3.106)	1.268(0.221-7.262)	2.200(0.467-10.358)	0.726(0.350-1.503)	0.538(0.227-1.273)	0.454(0.049-4.234)	1.279(0.310-5.282)	1.733(0.696-4.315)	0.973(0.426-2.224)	1.871(0.745-4.695)
aSM IgM	0.632(0.239-1.671)	0.391(0.047-3.254)	3.228(0.618-16.857)	1.159(0.217-6.194)	0.989(0.479-2.043)	0.921(0.409-2.076)	0.688(0.074-6.367)	0.785(0.159-3.872)	1.291(0.499-3.338)	1.166(0.505-2.693)	2.203(0.870-5.578)
aPA IgG	**3.026(1.066-8.593)**	0.520(0.131-2.069)	0.753(0.132-4.293)	0.718(0.131-3.938)	1.273(0.631-2.568)	1.959(0.835-2.276)	0.636(0.105-3.860)	4.071(0.495-33.512)	1.053(0.399-2.780)	0.416(0.183-0.946)	1.250(0.452-3.457)
aPA IgM	1.211(0.554-2.651)	0.637(0.146-2.781)	1.731(0.335-8.955)	1.436(0.327-6.312)	1.159(0.610-2.204)	0.631(0.299-1.329)	1.985(0.365-10.794)	0.968(0.256-3.661)	1.373(0.570-3.304)	0.761(0.345-1.677)	1.503(0.617-3.664)
aPS/PT IgG	1.866(0.828-4.206)	1.301(0.293-5.776)	0.878(0.150-5.141)	0.844(0.150-4.739)	1.603(0.821-3.131)	1.510(0.727-3.135)	0.684(0.109-4.306)	0.528(0.105-2.651)	1.590(0.654-3.867)	0.490(0.210-1.142)	1.392(0.562-3.449)
aPS/PT IgM	**2.537(1.101-5.848)**	0.469(0.110-1.993)	0.402(0.071-2.283)	1.143(0.258-5.067)	0.938(0.500-1.759)	1.552(0.760-3.169)	0.386(0.064-2.324)	1.463(0.388-5.522)	1.828(0.727-4.599)	1.163(0.539-2.509)	2.873(1.057-7.808)

Odds ratios (ORs) with 95% confidence intervals (CIs) are shown. Significant results (p<0.05) are marked in bold. Multivariable analysis has been adjusted for age and gender.

**Supplemental Figure 2** showed the detailed correlations among aPLs and diagnostic clinical manifestations, with the non-significant Pearson correlation coefficient (-0.1 ≤ r ≤ 0.1) crossed off. Pregnancy morbidity was associated with aSM IgM and aSM IgG. Pre-eclampsia or premature birth was associated with aSM IgG, aPI IgG, aPC IgG, and aPG IgG. Overall arterial events were associated with aPI IgG, aPC IgG, aPG IgG, and aPA IgG. Stroke was associated with aPI IgG. Lower limb artery occlusion was associated with aPC IgG, aPE IgG, aPG IgG, and aPA IgG. For venous events, a clinical association was only observed in DVT with aPS/PT IgG.

Correlations between extra-criteria manifestations and aPLs in APS patients were calculated with odds ratio in **Table 4**. Microvasculopathy was significantly associated with aPC IgG (OR 2.227, 95% CI 1.015-4.886) and aPG IgG (OR 2.279, 95% CI 1.029-5.050). Thrombocytopenia was significantly associated with aCL IgG/M (OR 2.990, 95% CI 1.544-4.251), aβ2GpI IgG/M (OR 2.085, 95% CI 1.022-4.251), LA (OR 3.915, 95% CI 1.491-10.278), aPI IgG (OR 2.555, 95% CI 1.332-4.901), aPS/PT IgG (OR 2.146, 95% CI 1.113-4.135), and aPS/PT IgM (OR 3.310, 95% CI 1.718-6.375). Hemolytic anemia was associated with LA (OR 4.781, 95% CI 1.052-21.734), aPC IgM (OR 2.960, 95% CI 1.136-7.709, aPE IgM (OR 3.464, 95% CI 1.296-9.258), and aPG IgM (OR 3.073, 95% CI 1.165-8.108). Valve lesion was positively associated with aSM IgM (Fisher test p = 0.039), APS nephropathy was positively associated with aβ2GpI IgG/M (Fisher test p = 0.019), and aPC IgG (OR 3.797, 95% CI 1.076-13.392), livedo reticularis was positively associated with aPE IgM (OR 15.391, 95% CI 1.392-1370.195), while epilepsy was negatively associated with aPI IgM (OR 0.204, 95% CI 0.052-0.806).

**Table 4:** Clinical correlation between non-criteria aPLs and clinical manifestations (n = 177).

	Microvasculopathy	thrombocytopenia	Hemolytic anemia	Valve lesion	APS nephropathy	Livedo reticularis	Cognitive impairment	epilepsy
aCL IgG/M	1.884(0.824-4.309)	**2.990(1.544-5.788)**	1.312(0.491-3.501)	2.147(0.555-8.304)	4.225(0.881-20.264)	3.548(0.343-36.748)	0.533(0.115-2.482)	1.892(0.481-7.446)
aβ2GpI IgG/M	**5.505(1.575-19.234)**	**2.085(1.022-4.251)**	0.709(0.261-1.928)	5.140(0.639-41.366)	**Fisher p = 0.019**	Fisher p = 0.323	2.868(0.332-24.778)	4.236(0.523-34.200)
LA	2.486(0.684-9.030)	**3.915(1.491-10.278)**	**4.781(1.052-21.734)**	4.098(0.504-33.313)	2.068(0.238-17.931)	Fisher p = 0.585	Fisher p = 0.348	Fisher p = 0.125
aPC IgG	**2.227(1.015-4.886)**	1.483(0.796-2.763)	0.810(0.300-2.188)	1.782(0.535-5.932)	**3.797(1.076-13.392)**	4.151(0.496-34.762)	1.267(0.272-5.888)	1.270(0.363-4.446)
aPC IgM	1.147(0.494-2.660)	1.247(0.649-2.396)	**2.960(1.136-7.709)**	1.032(0.293-3.630)	0.788(0.201-3.086)	0.638(0.062-6.544)	0.375(0.044-3.217)	0.427(0.088-2.067)
aPI IgG	1.434(0.639-3.221)	**2.555(1.332-4.901)**	0.804(0.311-2.076)	1.417(0.406-4.953)	4.124(0.861-19.766)	1.532(0.205-11.447)	0.972(0.209-4.514)	1.861(0.472-7.327)
aPI IgM	0.901(0.408-1.993)	1.188(0.632-2.232)	2.769(0.877-8.743)	0.410(0.123-1.367)	0.882(0.262-2.969)	0.525(0.074-3.704)	0.848(0.181-3.971)	**0.204(0.052-0.806)**
aPE IgG	2.167(0.978-4.804)	1.488(0.797-2.778)	0.868(0.331-2.276)	1.931(0.578-6.450)	2.802(0.808-9.715)	2.663(0.301-23.550)	1.271(0.267-6.062)	0.739(0.206-2.657)
aPE IgM	0.837(0.309-2.266)	0.883(0.424-1.841)	**3.464(1.296-9.258)**	0.481(0.099-2.335)	0.716(0.145-3.547)	**15.391(1.392-170.195)**	1.425(0.252-8.049)	0.257(0.031-2.100)
aPE IgA	1.268(0.377-4.271)	2.031(0.783-5.267)	1.823(0.509-6.527)	Fisher p = 0.305	Fisher p = 0.365	Fisher p = 1.000	1.828(0.189-17.645)	0.531(0.061-4.659)
aPG IgG	**2.279(1.029-5.050)**	1.181(0.617-2.261)	0.661(0.224-1.951)	2.452(0.735-8.180)	2.509(0.754-8.346)	10.851(0.925-127.261)	1.764(0.376-8.268)	1.176(0.323-4.276)
aPG IgM	0.847(0.357-2.008)	1.257(0.659-2.398)	**3.073(1.165-8.108)**	0.555(0.142-2.167)	1.222(0.338-4.415)	0.892(0.084-9.469)	0.355(0.041-3.091)	0.349(0.072-1.692)
aPG IgA	0.369(0.044-3.058)	2.014(0.625-6.491)	1.467(0.282-7.633)	Fisher p = 0.604	Fisher p = 0.604	Fisher p = 1.000	Fisher p = 1.000	Fisher p = 1.000
aSM IgG	0.899(0.366-2.207)	0.576(0.281-1.183)	0.591(0.185-1.892)	0.786(0.201-3.076)	0.890(0.224-3.534)	0.502(0.048-5.297)	1.052(0.193-5.733)	0.551(0.113-2.681)
aSM IgM	0.526(0.187-1.477)	0.520(0.246-1.100)	0.459(0.126-1.667)	**Fisher p = 0.039**	0.622(0.129-3.002)	Fisher p = 0.334	Fisher p = 0.195	0.268(0.033-2.177)
aPA IgG	2.665(0.957-7.417)	1.547(0.774-3.089)	1.731(0.544-5.504)	1.223(0.313-4.775)	4.638(0.576-37.328)	Fisher p = 0.323	2.380(0.277-20.462)	1.907(0.395-9.211)
aPA IgM	0.864(0.379-1.966)	1.111(0.594-2.077)	2.080(0.799-5.413)	0.789(0.225-2.770)	0.520(0.133-2.037)	0.427(0.041-4.403)	0.650(0.121-3.504)	0.521(0.130-2.081)
aPS/PT IgG	2.118(0.942-4.764)	**2.146(1.113-4.135)**	1.398(0.528-3.702)	2.008(0.605-6.665)	3.141(0.896-11.008)	0.679(0.088-5.257)	1.432(0.295-6.942)	2.714(0.775-9.503)
aPS/PT IgM	1.922(0.851-4.344)	**3.310(1.718-6.375)**	1.557(0.584-4.155)	4.344(0.915-20.610)	1.317(0.391-4.438)	1.193(0.167-8.503)	1.188(0.253-5.576)	3.862(0.804-18.564)

Odds ratios (ORs) with 95% confidence intervals (CIs) are shown. Significant results (p<0.05) are marked bold. Multivariable analysis has been adjusted for age and gender.

## Discussion

In patients under the age of 50, APS contributes to a significant part of recurrent reproduction losses as well as cerebro- or cardiovascular accidents (10). It has been postulated that the pathological features of APS are driven by intracellular signaling pathways in various cellular subtypes activated by criteria and non-criteria aPLs (11). Currently, a wide spectrum of aPLs has been discovered. They could directly bind to negatively charged phospholipids (e.g., aPG, aPI) or react with phospholipid-binding proteins (e.g., aPS/PT, aDM1) (12). In this study, we explored the diagnostic power and clinical significance of 16 non-criterial aPLs in APS patients.

Our results suggest that although a single aPL isotype may not have reached comparable diagnostic performance with aCL or aβ2GPI, the combinational test of aPC IgG/M, aPE IgG/M/A, aPI IgG/M, aSM IgG/M, and aPA IgG/M compared to aCL or aβ2GPI IgG/M only could significantly increase diagnostic sensitivity and accuracy. The titer of most aPLs shown for each group was also significantly higher in APS patients compared to disease or healthy controls. The good diagnostic values of aPC and aSM were consistent with our previous findings, while PE had higher sensitivity in this study (8). Volkov et al. also reported that aPI was more prevalent among APS patients compared to healthy and diseased control subjects with sepsis, but did not report a significantly higher level of aPE positivity (13). Nevertheless, since previous works did not calculate and compare the additional value of aPLs to criteria biomarkers, the results should be cautiously interpreted. Utilizing this method of comparison, we found that the detection of aCL IgA, ab2GPI IgA, aAnxV IgG/M, and aPS/PT IgG/M provided additive power (14). The introduction of these aPLs into routine laboratory practice could accelerate APS diagnostics.

In our 9 SNAPS patients, 8 (88.9%) were positive for at least one non-criteria aPLs, of which 3 patients had 5 or more positive aPLs. The most encountered aPLs were aPS/PT IgM (in 6 patients), followed by aPI IgG (in 5 patients) and aPA IgG (in 4 patients). In a USA/UK study including 68 SNAPS patients, Zohoury et al. found that 36.8% could be identified by 11 non-criteria aPLs, of which 11.8% were positive for aPS/PT (15). Litvinova and colleagues reported that 52.9% of 17 SNAPS patients could be identified by at least one of 18 non-criteria aPLs (16). Trugliia et al. documented that 81.9% of 61 female SNAPS patients with reproductive complications were positive with at least one of 5 new aPLs (17). In all, non-criteria aPLs could also provide additive value for the identification of SNAPS patients.

From the perspective of clinical relevance, we found that certain non-criteria aPLs are associated with both criteria and extra-criteria clinical manifestations. For diagnostic manifestations, our correlation analysis indicated that aSM IgG and IgM were positively associated with pregnancy morbidities, among which pre-eclampsia or premature birth was significantly associated with aSM IgG. Antibodies against SM were less reported in previous literature, which reside in the outer leaflet of the plasma membrane (12). Additionally, aPI IgG correlated with pre-eclampsia or premature birth, and it was also significantly correlated with stroke. Castanon et al. observed a high sensitivity for aPI (41.2%-59.2%) across manifestations including thrombotic and obstetric events (18). Our previous results also demonstrated a correlation between aPI IgG and thrombosis (8).

For other arterial events, we found that IgG of aPC, aPE, aPG, and aPA were associated with lower limb artery occlusion. Both PC and PE are major neutral components of the phospholipid, and their antibodies have been observed in pediatric patients with cerebral infarction (19). Several studies have reported that aPE was significantly associated with major clinical events including fetal loss and/or thrombosis, especially in the absence of laboratory APS tests (20). For venous events, we calculated the correlation of aPLs with pulmonary embolism, portal thrombosis, and cerebral and deep venous thrombosis. Only aPS/PT IgG positivity was associated with DVT. Several studies have supported that aPS/PT IgG was a strong indicator of the risk of thrombosis or obstetric complications and could be used as a confirmatory diagnostic marker (6, 7, 16). In addition, we did not find any clinical association for aPG or aPE IgA, which also had low sensitivity in APS diagnosis. IgA isotypes of non-criteria aPLs have hardly been investigated in previous studies, and more experience must be accumulated to further evaluate their ability in diagnosis.

Extra-criteria APS features could be associated with an increased risk of relapse and the need for additional therapies (21). Thus, the predictive value of non-criteria aPLs for these manifestations was explored. For microvasculopathy, aPC IgG and aPG IgG showed a significantly increased risk. Diffuse thrombotic microvasculopathy was a critical characteristic of catastrophic APS (CAPS) (22). Although we did not include these patients, the positivity of aPG IgG has been observed in 2 of 3 CAPS subjects previously (16). For other extra-criteria manifestations, many of these new aPLs presented a significant association with hematological disorders (thrombocytopenia or hemolytic anemia). One potential mechanism of thrombocytopenia was due to increased activation and destruction of platelets by aPLs (23). Activation of the complement pathway and coagulation system by aPLs was also hypothesized to be the underlying cause of hemolytic anemia (24).

Another interesting finding was a strong positive association between aPE IgM and livedo reticularis, which was observed to be linked with thrombosis and heart valve disease (25, 26). Additionally, heart valve lesions were positively associated with aSM IgM (by Fisher’s test), and APS nephropathy was positively associated with aPC IgG. While SM was found to be correlated with atherosclerosis and thrombosis (27), more subjects should be added to consolidate our findings. In summary, examination of the broad spectrum of non-criteria aPLs may allow better characterization of APS pathophysiology and multifaceted clinical phenotypes, which could shed light on the early diagnosis and better management of the disease.

This study has its limitations. Since most subjects were recruited from the outpatient clinic, the number of CAPS or severe patients could be unbalanced. Other promising biomarkers such as the anti-first domain (DI) of β2GPI were not explored here. The follow-up information of included patients was not sufficient for the surveillance of recurrent events, especially for thrombosis. Currently, the lack of standardized detection systems and their cut-off values may reduce the reliability of the findings.

## Conclusion

In conclusion, additional detection of non-criteria aPLs including aPC IgG/M, aPE IgG/M/A, aPI IgG/M, aSM IgG/M, and aPA IgG/M could assist in APS diagnosis. The positivity of certain aPLs was statistically associated with both criteria and extra-criteria APS clinical manifestations.

## Data availability statement

The original contributions presented in the study are included in the article/**Supplementary Material**. Further inquiries can be directed to the corresponding authors.

## Ethics statement

The studies involving human participants were reviewed and approved by the Ethics Committee of Peking Union Medical College Hospital. The patients/participants provided their written informed consent to participate in this study.

## Author contributions

All authors were involved in the design of this study. SL, YB, JM, QW, XT, ML, XZ, JZ, and CH contributed to the collection of blood samples and other experimental procedures. SL, YB, and JM were involved in data collection and pre-processing. SL and CH analyzed the data and wrote the manuscript. QW, XT, ML, XZ, and JZ contributed to the recruitment of patients and the evaluation of clinical data. All authors contributed to the article and approved the submitted version.

## References

[1] Miyakis S Lockshin MD Atsumi T Branch DW Brey RL Cervera R. International consensus statement on an update of the classification criteria for definite antiphospholipid syndrome (APS). J Thrombosis Haemostasis (2006) 4(2):295–306. doi: 10.1111/j.1538-7836.2006.01753.x 16420554

[2] Garcia D Erkan D. Diagnosis and management of the antiphospholipid syndrome. New Engl J Med (2018) 378(21):2010–21. doi: 10.1056/NEJMra1705454 29791828

[3] Hughes GR Khamashta MA. Seronegative antiphospholipid syndrome. Ann Rheumatic Diseases. (2003) 62(12):1127. doi: 10.1136/ard.2003.006163 PMC1754381 14644846

[4] Bradacova P Slavik L Ulehlova J Skoumalova A Ullrychova J Prochazkova J. Current promising biomarkers and methods in the diagnostics of antiphospholipid syndrome: A review. Biomedicines (2021) 9(2):166. doi: 10.3390/biomedicines9020166 PMC7914732 33567576

[5] Bertolaccini ML Amengual O Atsumi T Binder WL de Laat B Forastiero R. 'Non-criteria' aPL tests: Report of a task force and preconference workshop at the 13th international congress on antiphospholipid antibodies, Galveston, TX, USA, April 2010. Lupus. (2011) 20(2):191–205. doi: 10.1177/0961203310397082 21303836

[6] Shi H Zheng H Yin YF Hu QY Teng JL Sun Y. Antiphosphatidylserine/prothrombin antibodies (aPS/PT) as potential diagnostic markers and risk predictors of venous thrombosis and obstetric complications in antiphospholipid syndrome. Clin Chem Lab Med (2018) 56(4):614–24. doi: 10.1515/cclm-2017-0502 29166262

[7] Liu T Gu J Wan L Hu Q Teng J Liu H. "Non-criteria" antiphospholipid antibodies add value to antiphospholipid syndrome diagnoses in a large Chinese cohort. Arthritis Res Ther (2020) 22(1):33. doi: 10.1186/s13075-020-2131-4 32085759 PMC7035660

[8] Zhang S Wu Z Zhang W Zhang F Li Y Liu Y. Clinical performance of non-criteria antibodies to phospholipids in Chinese patients with antiphospholipid syndrome. Clinica Chimica Acta; Int J Clin Chem (2019) 495:205–9. doi: 10.1016/j.cca.2019.04.065 31002781

[9] Hochberg MC. Updating the American college of rheumatology revised criteria for the classification of systemic lupus erythematosus. Arthritis Rheumatol (1997) 40(9):1725. doi: 10.1002/art.1780400928 9324032

[10] Schreiber K Sciascia S de Groot PG Devreese K Jacobsen S Ruiz-Irastorza G. Antiphospholipid syndrome. Nat Rev Dis Primers (2018) 4:17103. doi: 10.1038/nrdp.2017.103 29321641

[11] Willis R Gonzalez EB Brasier AR. The journey of antiphospholipid antibodies from cellular activation to antiphospholipid syndrome. Curr Rheumatol Rep (2015) 17(3):16. doi: 10.1007/s11926-014-0485-9 25761923

[12] McIntyre JA Wagenknecht DR Faulk WP. Antiphospholipid antibodies: discovery, definitions, detection and disease. Prog Lipid Res (2003) 42(3):176–237. doi: 10.1016/S0163-7827(02)00048-6 12689618

[13] Volkov I Seguro L Leon EP Kovács L Roggenbuck D Schierack P. Profiles of criteria and non-criteria anti-phospholipid autoantibodies are associated with clinical phenotypes of the antiphospholipid syndrome. Auto- Immun highlights. (2020) 11(1):8. doi: 10.1186/s13317-020-00131-3 32467748 PMC7229627

[14] Hu C Li S Xie Z You H Jiang H Shi Y. Evaluation of the diagnostic value of non-criteria antibodies for antiphospholipid syndrome patients in a Chinese cohort. Front Immunol (2021) 12:741369. doi: 10.3389/fimmu.2021.741369 34567005 PMC8461188

[15] Zohoury N Bertolaccini ML Rodriguez-Garcia JL Shums Z Ateka-Barrutia O Sorice M. Closing the serological gap in the antiphospholipid syndrome: The value of "Non-criteria" antiphospholipid antibodies. J Rheumatol (2017) 44(11):1597–602. doi: 10.3899/jrheum.170044 28864642

[16] Litvinova E Darnige L Kirilovsky A Burnel Y de Luna G Dragon-Durey MA. Prevalence and significance of non-conventional antiphospholipid antibodies in patients with clinical APS criteria. Front Immunol (2018) 9:2971. doi: 10.3389/fimmu.2018.02971 30619328 PMC6302212

[17] Truglia S Capozzi A Mancuso S Recalchi S Spinelli FR Perricone C. A monocentric cohort of obstetric seronegative anti-phospholipid syndrome. Front Immunol (2018) 9:1678. doi: 10.3389/fimmu.2018.01678 30079071 PMC6062588

[18] Castanon A Pierre G Willis R Harris EN Papalardo E Romay-Penabad Z. Performance evaluation and clinical associations of immunoassays that detect antibodies to negatively charged phospholipids other than cardiolipin. Am J Clin Pathology. (2018) 149(5):401–11. doi: 10.1093/ajcp/aqy003 PMC5889035 29547897

[19] Korematsu S Yamada H Miyahara H Ihara K. Increased levels of anti-phosphatidylcholine and anti-phosphatidylethanolamine antibodies in pediatric patients with cerebral infarction. Brain Dev (2017) 39(6):542–6. doi: 10.1016/j.braindev.2017.01.010 28238458

[20] Pignatelli P Ettorre E Menichelli D Pani A Violi F Pastori D. Seronegative antiphospholipid syndrome: refining the value of "non-criteria" antibodies for diagnosis and clinical management. Haematologica (2020) 105(3):562–72. doi: 10.3324/haematol.2019.221945 PMC7049333 32001534

[21] Guédon AF Catano J Ricard L Laurent C de Moreuil C Urbanski G. Non-criteria manifestations in primary antiphospholipid syndrome: a French multicenter retrospective cohort study. Arthritis Res Ther (2022) 24(1):33. doi: 10.1186/s13075-022-02726-9 35078523 PMC8788111

[22] Bucciarelli S Cervera R Espinosa G Gómez-Puerta JA Ramos-Casals M Font J. Mortality in the catastrophic antiphospholipid syndrome: causes of death and prognostic factors. Autoimmun Rev (2006) 6(2):72–5. doi: 10.1016/j.autrev.2006.06.007 17138246

[23] Artim-Esen B Diz-Küçükkaya R İnanç M. The significance and management of thrombocytopenia in antiphospholipid syndrome. Curr Rheumatol Rep (2015) 17(3):14. doi: 10.1007/s11926-014-0494-8 25740703

[24] Ames PRJ Merashli M Bucci T Pastori D Pignatelli P Arcaro A. Antiphospholipid antibodies and autoimmune haemolytic anaemia: A systematic review and meta-analysis. Int J Mol Sci (2020) 21(11):4120. doi: 10.3390/ijms21114120 PMC7313475 32527000

[25] Kontic M Stojanovich L Mijailović-Ivković M Velinović M Srnka J Zdravkovic M. Are the cutaneous manifestations in patients with primary antiphospholipid syndrome a marker for predicting lung manifestations? Clin Exp Rheumatol (2018) 36(1):56–61. 28770705

[26] Francès C Niang S Laffitte E Pelletier F Costedoat N Piette JC. Dermatologic manifestations of the antiphospholipid syndrome: Two hundred consecutive cases. Arthritis Rheumatol (2005) 52(6):1785–93. doi: 10.1002/art.21041 15934071

[27] Kikas P Chalikias G Tziakas D. Cardiovascular implications of sphingomyelin presence in biological membranes. Eur Cardiol (2018) 13(1):42–5. doi: 10.15420/ecr.2017:20:3 PMC6159463 30310470

