# Supplementary material for: Prevalence and diagnostic value of non-criteria antiphospholipid antibodies for antiphospholipid syndrome in Chinese patients
Source: Front Immunol. 2023 Apr 12;14:1107510. doi: 10.3389/fimmu.2023.1107510 (PMC10132625; doi:10.3389/fimmu.2023.1107510)
Supplement: Supplementary file 1 [file DataSheet_1.docx]

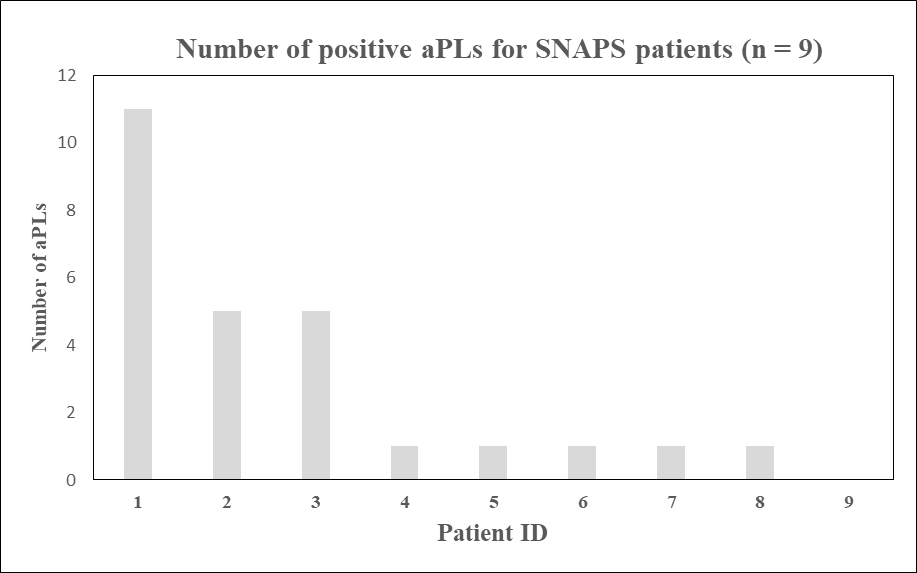


**Figure S1. Number of positive extra-criterial aPLs for SNAPS patients.**


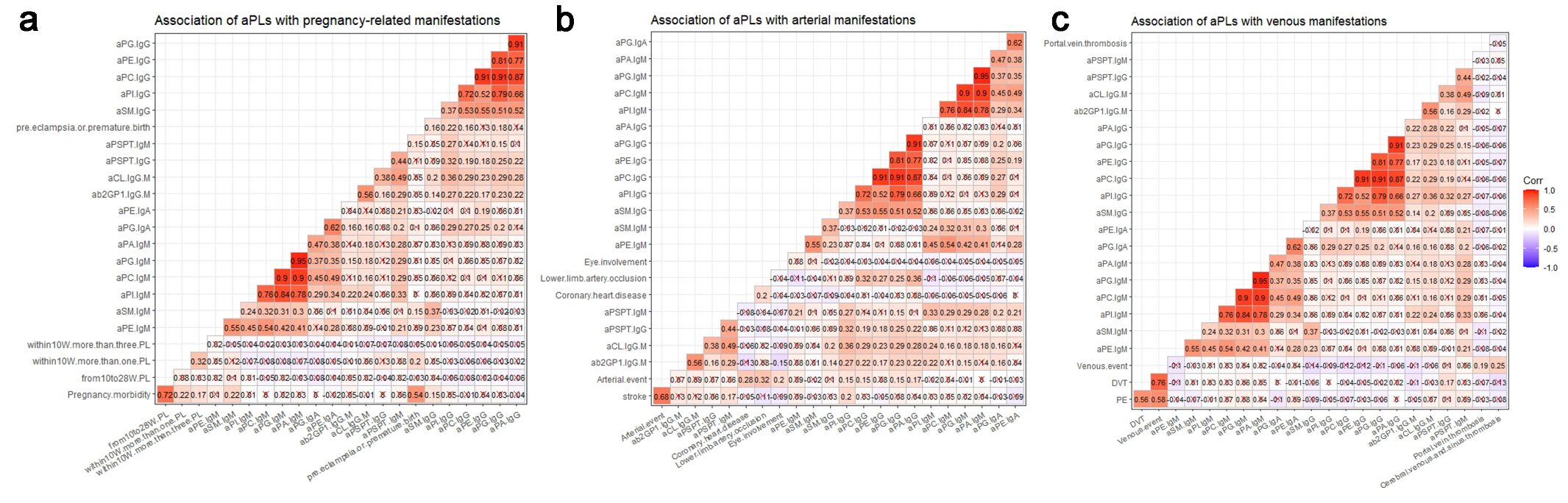


**Figure S2. The association of extra-criteria aPLs with diagnostic clinical manifestations (n = 177).**
